# Impact of the coronavirus pandemic on maxillofacial trauma: A retrospective study in southern Spain

**DOI:** 10.4317/medoral.25063

**Published:** 2022-04-03

**Authors:** Pedro Infante-Cossio, Macarena Fernandez-Mayoralas-Gomez, Luis Miguel Gonzalez-Perez, Rafael Martinez-de-Fuentes, Angel Rollon-Mayordomo, Eusebio Torres-Carranza

**Affiliations:** 1 Department of Surgery, School of Medicine, University of Seville, Seville, Spain; 2 Department of Oral and Maxillofacial Surgery, Virgen del Rocio University Hospital, Seville, Spain; 3 Department of Stomatology, School of Dentistry, University of Seville, Seville, Spain

## Abstract

**Background:**

The coronavirus pandemic has impacted health systems worldwide, with Spain being one of the most affected countries. However, little is known about the extent to which the effects of staying home, social distancing, and quarantine measures have influenced the epidemiology of patients with maxillofacial trauma. The aim of this study was to analyze the impact of the coronavirus pandemic on the incidence, demographic patterns, and characteristics of maxillofacial fractures in the largest hospital in southern Spain.

**Material and Methods:**

Data from patients who underwent surgery for maxillofacial fractures during the first year of the pandemic between 16 March 2020 and 14 March 2021 (pandemic group) were retrospectively compared with a control group during the equivalent period of the previous year (pre-pandemic group). The incidence was compared by weeks and by lockdown periods of the population. Demographic information, aetioloy, fracture characteristics, treatment performed, and days of preoperative stay were evaluated. Descriptive and bivariate statistics were calculated (*p*<0.05).

**Results:**

During the first year of the pandemic, there was a 35.2% reduction in maxillofacial fractures (n=59) compared to the pre-pandemic year (n=91, *p*=0.040). A significant drop was detected during the total home lockdown period of the population (*p*=0.028). In the pandemic group, there was a reduction in fractures due to interpersonal aggressions, an increase in panfacial fractures, a significant increase in other non-facial injuries associated with polytrauma (*p*=0.037), a higher number of open reduction procedures with internal fixation, and a significantly longer mean preoperative stay (*p*=0.016).

**Conclusions:**

The first pandemic year was associated with a decline in the frequency of maxillofacial trauma and a change in the pattern and characteristics of fractures. Inter-annual epidemiological knowledge of maxillofacial fractures may be useful for more efficient planning of resource allocation and surgical practice strategy during future coronavirus outbreaks and population lockdowns.

** Key words:**SARS-CoV-2, coronavirus infection, pandemic, maxillofacial trauma, Spain.

## Introduction

The outbreak of the new coronavirus disease (COVID-19), initially identified in December 2019 in the People's Republic of China (1), quickly spread to several countries around the world, with Spain being one of the most affected. On 31 January 2020, the first confirmed case of severe acute respiratory syndrome due to coronavirus-2 (SARS-CoV-2) was notified in Spain (2). From then on, an unprecedented epidemiological situation emerged that led the government to enact drastic health measures to reduce community transmission.

The first state of health alarm in Spain, announced on 14 March 2020, determined the total quarantine of the population in their homes and the interruption of non-essential activities. Despite this, the great expansion of the pandemic during the first year and the successive waves of COVID-19 infections forced the Spanish authorities to decree prolonged periods of confinement, restriction of movements, activities, sports and travels, bans on social outings and meetings, multiple curfews, and obligation of social distancing and facial mask. During the first year of the pandemic, three consecutive Royal Decrees declaring an extended state of alarm were proclaimed in Spain that caused an extraordinary disruption of normal hospital activities, decreasing patient admissions to emergency departments, with delays in patient medical care and redistribution of emergency and elective surgeries.

The coronavirus pandemic is requiring an enormous effort from world hospitals that treat maxillofacial trauma. A global survey evaluating oral and maxillofacial surgery (OMFS) service delivery during the outbreak found that one of the most maintained services was trauma, according to 82% of centers worldwide (3). In the Spanish National Health System, an observational study carried out in a tertiary hospital described a significant reduction in the total number of visits to a general trauma emergency department in 2020 (4). However, there are no data on the relative impact on the medical care of patients with maxillofacial trauma, as COVID-19 is an unexpected new disease (5), and therefore little is known about the extent to which the effects of staying home, social distancing, and quarantine measures have influenced patient management and surgical delays.

This study aimed to evaluate the impact of the COVID-19 pandemic on maxillofacial trauma at the Virgen del Rocio University Hospital. This hospital is the largest in southern Spain, serving more than 300,000 emergencies in 2019, of which approximately 70,000 corresponded to trauma. The institution has emergency coverage for patients with severe acute trauma in a potential area of 1.45 million residents. The hypothesis of this study was that the measures ordered by the government of Spain and regional authorities during the first year in response to the COVID-19 pandemic had a significant influence on the incidence and characteristics of patients operated on by maxillofacial trauma. The objective was to determine the inter-annual variation in the incidence, demographic patterns, and characteristics of maxillofacial fractures to identify areas for improvement and provide recommendations on a more efficient planning of resources and a surgical practice strategy that may be useful in the event of new coronavirus outbreaks and further population lockdowns.

## Material and Methods

- Study design, sample, and setting. This study consisted of a retrospective comparative cohort study of patients operated on for maxillofacial fractures at the OMFS Department of the Virgen del Rocio University Hospital in Seville (Spain). The incidence and characteristics of the patients during the first year of the coronavirus pandemic were compared with a control group from the equivalent period of the previous year. In accordance with the above, two groups were established as follows: 1) pandemic group (PG) or experimental group: between 16 March 2020 and 14 March 2021 (52 weeks); 2) pre-pandemic group (PPG) or control group: between 18 March 2019 and 15 March 2020 (52 weeks).

Inclusion criteria were: 1) patients 14 years or older operated under general anesthesia for one or more maxillofacial fractures and 2) having complete clinical data recorded in their medical histories. Exclusion criteria were: 1) patients with maxillofacial trauma treated outpatiently or under local anesthesia/sedation, 2) isolated nasal bone fractures and dental fractures, and 3) interventions for post-traumatic complications, sequelae of previous trauma, or removal of osteosynthesis plates. The study protocol was approved by the Hospital’s Ethics Committee (Internal Code: 0975-N-21) and followed the Declaration of Helsinki on medical protocol and ethics.

The main variable was the evaluation of maxillofacial fractures operated during the first year of the pandemic (PG) and the equivalent period of the previous year (PPG). The mean incidence was compared by weeks and by periods of confinement and mobility restriction. The periods declared by the Government of Spain and the regional authorities were divided into three phases: 1) total restriction of mobility (home lockdown), 2) partial restriction of mobility (municipal, provincial, and regional perimetral lockdown), and 3) no mobility restriction (de-escalation). In all phases, there were curfews, limitations on social meetings, social distancing measures, and mandatory use of facial masks. In the PG, the total number of hospitalized patients with COVID-19 was graphically compared with the weekly incidence of fractures.

Demographic and clinical variables were collected from the medical history of each patient along with their orthopantomography and computed tomography (CT), where appropriate. We collected the following data: demographic information (age, sex), aetiology (road traffic accident, interpersonal aggression, fall, sports accident), anatomical location (mandible, maxilla, orbito-zygomatic, panfacial), other associated non-facial injuries, patient referral (from the emergency department, from another centre), radiographs (orthopantomography, CT), type of treatment (maxillomandibular fixation screws, open reduction with internal fixation -ORIF- with or without maxillomandibular fixation screws, closed reduction by Gillie’s temporal approach), and days of preoperative stay.

- Surgical management during the coronavirus pandemic. Following the recommendations of the Spanish Society of OMFS on the management of patients during the COVID-19 pandemic, maxillofacial procedures were considered a specific source of risk of transmission of contagion due to the generation of aerosols and proximity to the patient's mouth. Consequently, cases of maxillofacial trauma were reconsidered as a non-delayed emergency. As a rule, initially all patients were assumed to be potentially infectious. Within 24 hours before surgery, a polymerase chain reaction (PCR) screening test for SARS-CoV-2 infection was performed. Patients would be operated on only if they had negative PCR results and had not been in close contact with confirmed cases, showed no symptoms suggesting possible COVID-19 infection, and had no travel history within 14 days. Since all documented cases in the current study showed negative results for COVID-19 tests, procedures were performed under general anesthesia in a specific operating room with primary protection of personnel for clear COVID-19 patients. In patients with positive PCR, treatment would be postponed for a minimum of two weeks and the whole process would then be repeated.

- Statistical analysis. Data were tabulated in a Microsoft Excel spreadsheet (Microsoft Corporation, Redmond, WA, USA) without recognizing the patient’s identity, only accessible to the main researcher. Both the principle of patient autonomy and their confidentiality were respected at all times of the study. A comparison between groups was made to evaluate the association between the incidence per week and per period of confinement. The outcome variables were analyzed using the SPSS statistical program, version 25 (IBM Corporation, Armonk, NY, USA). The chi-square test and the independent samples t test were used to make statistical inferences and generate the corresponding Figures. *p* values less than 0.05 were considered statistically significant.

## Results

**Figure 1:**
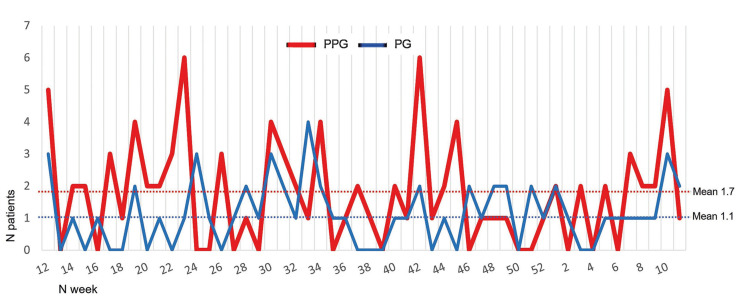
Patients operated on for maxillofacial trauma in the PPG and PG by weeks.

**Figure 2:**
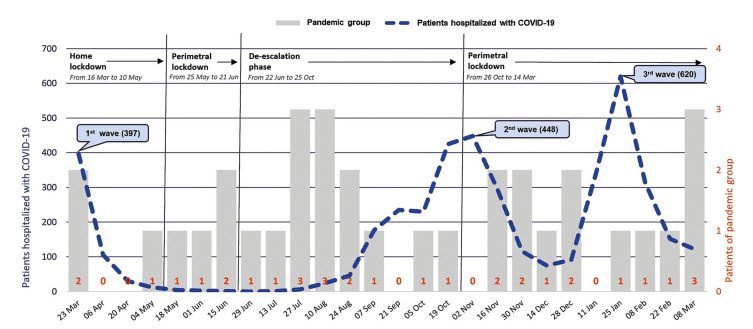
Comparison between patients operated on for maxillofacial trauma in the PG and the total number of hospitalized patients with COVID-19.

A total of 150 patient medical records were analysed, of which 91 cases were operated on in the pre-pandemic year and 59 cases in the pandemic year (a gross reduction of 35.2%). During the pre-pandemic year, 1.7 cases/week were operated, and in the pandemic year, 1.1 cases/week (*p*=0.040) (Table 1). In the PG, the greatest reduction occurred between weeks 12 and 19, corresponding to the total home lockdown of the population in April and May 2020 (Fig. 1). The analysis of incidence according to the confinement periods of the population and mobility restrictions showed a significant decrease in the volume of fractures in the total phase of mobility restriction (home lockdown) (Table 1) (*p*=0.028). Fig. 2 graphically compares the weekly evolution of the total number of patients hospitalized with COVID-19 with patients operated on for maxillofacial trauma. A decrease in cases was observed as the number of patients admitted with COVID-19 increased in each of the three waves of contagion in Spain.

Table 2 shows the characteristics of the patients included in this study. In the PPG, the mean age was 36.9 years (range 14-86) and in the PG 32.8 years (range 15-67) (*p*=0.124). The most frequent age group was between 20 and 39 years of age (47.3% in the PPG and 49.2% in the PG) (*p*=0.406). Most of the patients were male (79.1% in the PPG and 83.1% in the PG) (*p*=0.673). The male/female ratio was 3.8 and 4.9 in the PPG and PG, respectively. In the PPG, the main aetiologies were falls (36.3%) and interpersonal aggressions (35.2%), while in the PG there were falls (33.9%) and road traffic accidents (33.9%) (*p*=0.420). The most frequent anatomical location was the mandible (60.4% in the PPG and 54.2% in the PG) (*p*=0.706). In the PG, a higher percentage of panfacial fractures (20.3% in the PG vs 13.2% in the PPG) was diagnosed. In the PG, a significant relative increase was observed in the percentage of patients with other non-facial injuries associated with polytrauma (28.8% in the PG vs 14.3% in the PPG) (*p*=0.037).

Table 3 reveals an overview of patient management. Most of the patients were admitted through the emergency department (50.5% in the PPG and 55.9% in the PG) (*p*=0.616). In the PPG, CTs were performed in 66 patients (72.5%) while in the PG in 50 patients (84.7%) (*p*=0.059). In both groups, surgical procedures by ORIF with or without maxillomandibular fixation screws predominated (80.2% in the PPG and 88.1% in the PG, *p*=0.369). The mean days of preoperative stay were significantly longer in the PG (10.9 days, range=2-55) compared to the PPG (7.8 days, range=1-32) (*p*=0.016).

**Table 1:**
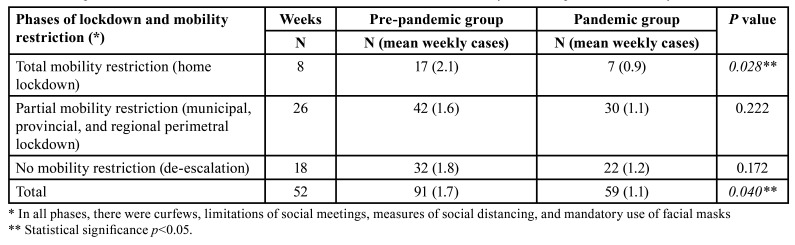
Comparison of the incidence of maxillofacial trauma between the PPG and PG by lockdown phases and mobility restriction.

**Table 2:**
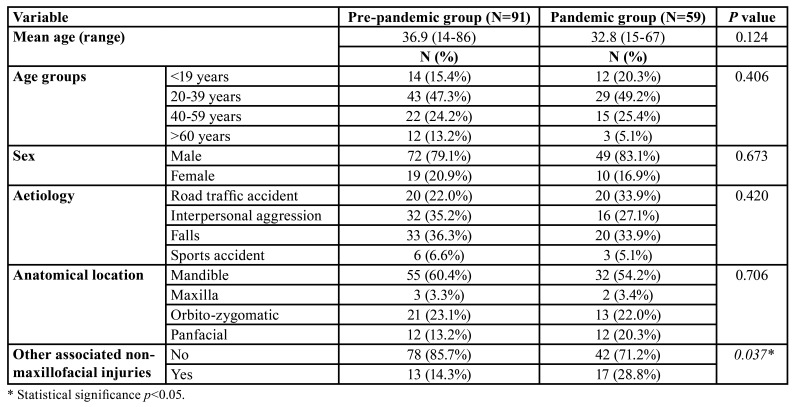
Patient characteristics.

**Table 3:**
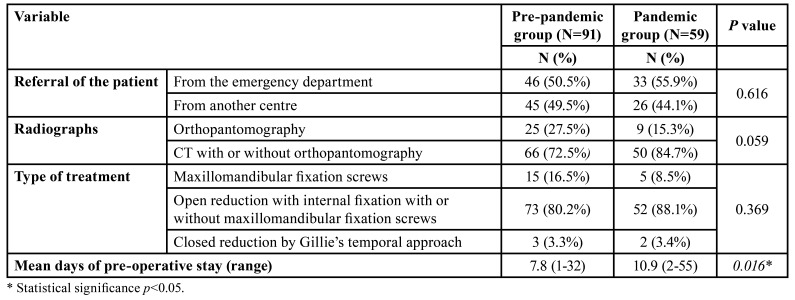
Overview of patient management.

## Discussion

The data obtained in this study revealed a significant decrease in the incidence of maxillofacial trauma during the first year of evolution of the COVID-19 pandemic in southern Spain. As expected, our findings indicated a gross drop of 35.2% in the total volume of cases with a significant decrease in the weekly incidence from 1.7 cases/week to 1.1 cases/week (*p*=0.040). We chose to compare an equivalent period of one year on a weekly basis to avoid inter-seasonal variations since the epidemiology of trauma can fluctuate from month to month. A significant reduction in the months of April and May 2020 was found, coinciding with the phase of total home lockdown of the non-essential population. Furthermore, our study graphically disclosed a drop in the number of fractures coinciding with the increase in hospitalized patients with COVID-19 during the three waves declared in the first year of the pandemic.

Several studies have reported about the influence of the COVID-19 outbreak on the frequency of maxillofacial fractures. To our knowledge, this is the only epidemiological study conducted in Spain that objectively evaluates the variation in the incidence of maxillofacial trauma, taking into account the multiple phases of population mobility restrictions. Furthermore, this comparative study considered a full year of pandemic by evaluating the association of clinical and therapeutic variables. Although our findings are difficult to contrast with other previous reports due to the heterogeneity of the studies, with different time periods and disparity in the restriction measures applied according to each country, all studies indicated a clear decrease in cases of maxillofacial trauma (6-18).

In general terms, although the reduction in the annual number of maxillofacial fractures was the most evident effect resulting from the measures taken to Fight against the pandemic, patient characteristics and fracture patterns also exhibited relevant changes. The gross and relative comparison of the demographic variables revealed that the mean age fell 4.1 years (reduction of 11.2%) and the number of males increased 5.1% in the first pandemic year (12). These data suggested that younger male adults behaved more actively during the pandemic year, being more exposed to trauma compared to older people.

The most common cause of fractures in both groups was falls, as previously reported in other studies (11,17). Interestingly, in the pandemic year, road traffic accidents increased while interpersonal assaults decreased. These etiological changes responded to multiple factors. Presumably, the decrease in interpersonal aggressions was related to changes in human behaviour motivated by social distancing measures and the prohibition of social meetings and nightlife (12,19). However, the relative increase in road traffic accidents in times of restrictions on population movement is striking. Probably, these accidents occurred in displacements for work needs in the essential population or for reasons of absolute emergency or health in the non-essential population.

In the first year of the pandemic, the percentage of mandibular fractures declined while panfacial fractures increased (11). These data can be explained by the relative increase in complex accidents together with the relative decrease in interpersonal aggressions. Furthermore, these data are related to the significant increase in the percentage of patients with other non-facial injuries associated with polytrauma and the fact that patients were originally referred to the emergency department with severe acute trauma, without having previously visited other lower-level centres (18).

Several OMFS practice guidelines recommended a more conservative therapeutic approach during the pandemic outbreak to simplify surgical interventions, reduce operating times, minimize pressure on hospital resources, and avoid potential exposure of healthcare workers to coronavirus by the generation of high-risk aerosols (7,8,13,18,20). Additionally, trauma cases that can wait more than a week have been recommended to be treated as non-delayed emergencies or elective cases (21). However, this was not the case in our clinical setting. We did not change our usual surgical management strategy because none of the patients tested positive for COVID-19 upon admission or developed an infection during their hospital stay. In the PG, relatively more ORIF procedures were performed while conservative treatments such as maxillomandibular fixation screws were reduced, probably due to the increase in panfacial fractures and their complexity. Similarly, 16.8% more preoperative CTs were requested for diagnosis, which can also be explained by the complexity of the fractures.

Despite the fact that the COVID-19 pandemic caused a global reduction in the number of trauma patients, our department remained operational due to the redistribution of hospital infrastructure and personnel and the modification of the patient circuit. All cases documented in the current study were able to be operated in a clear COVID-19 operating room specifically equipped for the OMFS Department because they had negative test results. The data from our study indicated a significant increase in the mean days of preoperative stay, growing by 3.1 days in the PG. This may be explained by the significant number of patients who presented other injuries associated with multiple traumas, mainly orthopaedics, and delays in surgical scheduling, redistribution of operating room resources, and the waiting period to perform a screening test for SARS-CoV-2 infection.

In general, the data from our study suggested that the reduction in the volume of maxillofacial trauma and changes in clinical and epidemiological patterns were related to mobility restrictions, social distancing measures, and curfews (10,16). Coronavirus may continue to infect for some time, so there is concern that new outbreaks will be repeated in the near future (22). As maxillofacial fractures are frequently referred to emergency departments around the world, essential primary services for trauma injuries must be maintained while the pandemic continues. For the most efficient planning of resource allocation, it will be crucial to know if the decrease in maxillofacial trauma will persist in the mid- and long-term and, more importantly, if the epidemiological situation will return to pre-pandemic trends. This inter-annual study of maxillofacial trauma may allow comparison with other studies to achieve a comprehensive perspective on the impact of the coronavirus pandemic.

This study presented some limitations that must be addressed. The main limitation was being a retrospective study in nature. However, since hospital medical records were recorded electronically, we believe our data set is accurate, valid, and complete. This is essentially a single-centre study, which may not be fully representative of the entire country’s trend, although our institution represents the hospital with the highest volume of trauma in southern Spain and one of the largest in our country. Finally, we compared the same period one year earlier to build the control cohort, so it is not known whether a different length period or conFigured in another way would have generated similar results. The strengths were based on the relatively high number of patients operated on by the same team following the same protocols and for a sufficiently long period of time (one year), which makes the results generalizable and comparable to other hospitals in Spain to achieve a comprehensive and complete perspective of the impact of the coronavirus pandemic on maxillofacial trauma in our country.

## Conclusions

This is the first study conducted in Spain that provides data on incidence and patterns of maxillofacial trauma during the national lockdown. Within the limitations of this retrospective study, it can be concluded that the measures taken to control the transmission of the COVID-19 pandemic had a significant impact on the epidemiology of maxillofacial fractures, resulting in a decrease in volume and a change in the pattern and characteristics of the patients. Furthermore, inter-annual epidemiological knowledge of maxillofacial trauma during the pandemic may be useful for more efficient planning of resource allocation in the future.
